# Exploring the impact of co‐created dementia education sessions delivered in‐person and online on dementia knowledge and attitudes

**DOI:** 10.1002/alz70860_101462

**Published:** 2025-12-23

**Authors:** Tahia Mujtaba, Gabriela E Caballero, Eman Shatnawi, Joyce Siette, Ann Dadich, Michelle DiGiacomo, Genevieve Z Steiner‐Lim, Diana Karamacoska

**Affiliations:** ^1^ Western Sydney University, Campbelltown, NSW, Australia; ^2^ NICM Health Research Institute, Western Sydney University, Sydney, NSW, Australia; ^3^ Western Sydney University, Sydney, NSW, Australia; ^4^ University of Technology Sydney, Sydney, NSW, Australia; ^5^ Translational Health Research Institute, Western Sydney University, Penrith, NSW, Australia

## Abstract

**Background:**

Many adults in Australia report limited knowledge about dementia and its causes, potentially perpetuating stigma towards the condition. Due to the ageing population, it is essential to address these knowledge gaps and promote positive attitudes towards individuals living with dementia. This study investigated whether co‐created dementia information sessions, delivered in‐person and online, impacted dementia knowledge and attitudes among adults.

**Method:**

This study was conducted with the South‐Western Sydney Dementia Network and the Canterbury‐Bankstown Dementia Alliance using community‐based participatory research methods. A two‐hour educational intervention addressed what dementia is, its causes, care strategies and local support services. It was delivered online, Australia‐wide, and in‐person across South‐Western Sydney, due to the region's increasing ageing population and dementia prevalence. A questionnaire was used to compare responses on ten items of the Dementia Knowledge Assessment Scale (DKAS) and 15 items of the Dementia Diagnosis Attitudes Scale (DDAS) from two intervention groups, in‐person and online, and a comparison group who received no education. Differences in mean ranks were compared and described statistically.

**Result:**

For nine of the ten DKAS items, the in‐person (*n* = 16) and online (*n* = 62) intervention groups demonstrated a greater tendency toward correct answers relative to the no education comparison group (*n* = 202), all *p* < .001. Only two of the 15 DDAS items showed statistically significant differences. The group that did not receive the intervention (*n* = 22) had more positive attitudes for these two items compared to the intervention groups (in‐person: *n* = 108; online: *n* = 14).

**Conclusion:**

Participants who received the intervention showed better dementia knowledge scores than the participants who did not receive it, regardless of the delivery mode. However, the single session was insufficient to meaningfully influence attitudes, emphasising the need for tailored sociocultural interventions to reduce public stigma against dementia.

